# Prolidase-proline oxidase axis is engaged in apoptosis induction by birch buds flavonol santin in endometrial adenocarcinoma cell line

**DOI:** 10.3389/fmolb.2023.1247536

**Published:** 2023-09-06

**Authors:** Lukasz Szoka, Jolanta Nazaruk, Joanna Giegiel, Valery Isidorov

**Affiliations:** ^1^ Department of Medicinal Chemistry, Medical University of Bialystok, Białystok, Poland; ^2^ Department of Pharmacognosy, Medical University of Bialystok, Białystok, Poland; ^3^ Institute of Forest Sciences, Białystok University of Technology, Białystok, Poland

**Keywords:** birch, santin, apoptosis, proline oxidase, prolidase

## Abstract

Cancer of the corpus uteri and cervix uteri, collectively ranks second among new cancer cases in women after breast cancer. Therefore, investigation of new anticancer agents and identifying new molecular targets presents a challenge to improve effectiveness of chemotherapy. In this study, antiproliferative activity of flavonoids derived from the buds of silver birch and downy birch was evaluated in endometrial cancer Ishikawa cells and cervical cancer HeLa cells. It was found that flavanol santin reduced viability of both cell lines better than other flavonoids, including apigenin and luteolin. Moreover, this activity was slightly higher than that induced by the chemotherapy drug, cisplatin. Santin promoted intrinsic and extrinsic apoptosis pathways in cancer cells, but it had low toxicity in normal fibroblasts. The mechanisms of impairing cancer cell viability included induction of oxidative proline catabolism, however in different ways in the cell lines used. In HeLa cells, increase of proline oxidation was due to activation of p53 leading to proline oxidase upregulation. In contrast, in Ishikawa cells, having basal proline oxidase level significantly higher than HeLa cells, santin treatment decreased its expression. Nevertheless, proline oxidation was induced in these cells since santin increased expression and activity of prolidase, an enzyme providing proline from protein degradation. In both cell lines, proline oxidation was associated with generation of reactive oxygen species leading to reduction in cell viability. Our findings reveal the involvement of proline oxidase in induction of apoptosis by santin and identify a role of prolidase in proline oxidase-dependent apoptosis.

## 1 Introduction

Natural product research is an important drug discovery strategy, offering a huge variety of scaffolds and structural complexity. It is estimated that only about 15% of the existing plant species have been phytochemically studied (Atanasov et al., 2015). This is further complicated by the uneven distribution of secondary metabolites in plant organs. Moreover, the nature of their distribution depends on both the stage of development and the age of the plants (Fico et al., 2000; Baraldi et al., 2008; Nešović et al., 2021).

Birch is one of the plants with great medicinal potential. The leaves, and less commonly the buds, of various birch species are used in folk medicine as a diuretic and an anti-inflammatory agent (European Medicines Agency, 2022). The genus *Betula* includes various types of trees and shrubs that are widely distributed in the northern hemisphere. Two birch species, silver birch (*Betula pendula* Roth) and downy birch (*Betula pubescens* Ehrh), grow throughout almost all of Eurasia (Dubois et al., 2020). Recently, we have shown that the buds of birches, in particular buds of downy birch, are a rich source of flavonoids and the composition of flavonoid fraction of buds and leaves differs significantly (Isidorov et al., 2021; Szoka et al., 2021).

Flavonoids are supposed to have a plethora of favourable effects in humans, such as anti-inflammatory, anti-aging, cardio-protective, neuroprotective, immunomodulatory, antidiabetic, antibacterial, antiparasitic and antiviral. In the last years anti-cancer activity of flavonoids was extensively studied providing new insights into their multi-targeted action (Dias et al., 2021; Bag et al., 2023).

**FIGURE 1:**
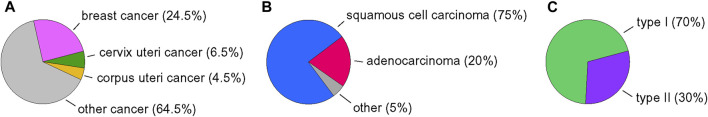
Overview of endometrial and cervical cancer statistics. **(A)** Incidence of selected cancers in women in 2020. **(B)** Subtypes of endometrial cancers. **(C)** Subtypes of cervical cancer. Data were collected from (Small et al., 2017; Sung et al., 2021; Zhou et al., 2023).

Cancer is a leading cause of death in most countries in the world. In 2020, about 600,000 new cases of cervix uteri cancer and 420,000 new cases of corpus uteri cancer were diagnosed worldwide. In total, this accounts for 11% of all cancer cases in women (Figure 1A) and ranks second among new cancer cases after breast cancer (24.5%) (Sung et al., 2021). Most cases of cervical cancer result from infection with the human papillomavirus (HPV); therefore, it is considered to be mostly preventable in future by HPV vaccination and screening programs (Cohen et al., 2019). Transformation of cells by HPV depends on viral oncoproteins E6 and E7. They inactivate p53 and retinoblastoma protein, respectively, leading to uncontrolled cell proliferation and increased resistance to apoptosis (Hengstermann et al., 2001; Singini et al., 2023). However, upregulation of human proto-oncogene MYC by integration of the HPV genome was also reported (Zhang et al., 2016). About 75% of cervical cancer cases are squamous cell carcinomas (Figure 1B). Adenocarcinomas account for approximately 20% (Small et al., 2017). Treatment options for cervical cancer include surgery, radiotherapy, and chemotherapy—mainly a combination of cisplatin and paclitaxel, supplemented as needed with anti-VEGF agent bevacizumab (Cohen et al., 2020). To date, inhibition of cell growth or induction of apoptosis by a few flavonoids (e.g., apigenin, chrysin, kaempferol and luteolin) were reported in cervical cancer and endometrial cancer cell lines (Csapi et al., 2010; Lei et al., 2019; Raina et al., 2021; Zhan and Wei, 2021; Liang et al., 2022).

Endometrial carcinomas were traditionally classified into two groups (Figure 1C). Type I (endometrioid type) is hormone-dependent with a favourable prognosis and represents 70% of newly diagnosed cases, however its grade 3 based on International Federation of Gynaecology and Obstetrics (FIGO) system (poorly differentiated cancer) is classified to second group. Type II (nonendometrioid type, high-grade) occurs in older patients and has a poorer prognosis. Molecular classification system divides endometrial cancers into groups regarding mutation status in gene encoding DNA polymerase epsilon (POLE), expression of MMR mismatch repair proteins, and expression of p53 (Crosbie et al., 2022). However, mutation in the tumour suppressing p53 gene is more frequent in type II (Baiden-Amissah et al., 2021). Therapy for endometrial cancer includes surgery, radiation therapy, and chemotherapy (the combination of carboplatin and paclitaxel) (Miller et al., 2020). Since p53-abnormal endometrial cancer shows an unfavourable prognosis and high disease-specific mortality, it is important to investigate efficient and relatively safe anticancer agents and to characterise novel molecular targets for cancer therapy.

Cancer cells are characterized by metabolic reprogramming resulting in increased synthesis of macromolecules and thereby increasing cancer biomass. On the other hand, rewiring of metabolism creates metabolic weakness that may provide a new approaches for cancer therapy. Modulation of amino acids metabolism is considered as a promising strategy for targeting cancer growth (Li and Zhang, 2023). Proline is an endogenous amino acid produced in the human body from glutamate and ornithine or recycled from proteins. Inside the cell, proline can be used for protein synthesis or oxidised to pyrroline-5-carboxylate and further to glutamate or ornithine. The first stage of proline oxidation is catalysed by mitochondrial proline oxidase (Cappelletti et al., 2018). Upregulation of proline catabolism is associated with apoptosis since p53 was found to strongly induce proline oxidase gene expression (Polyak et al., 1997). In this condition, proline oxidation is accompanied by massive generation of reactive oxygen species (ROS) and subsequently with induction of intrinsic and extrinsic apoptosis pathways (Liu et al., 2005; Liu et al., 2006; Hu et al., 2007). The role of protein breakdown in maintaining an adequate supply of proline for cells is not fully understood. Proteases involved in degradation of endogenous and dietary proteins are not capable of hydrolysing the imide bond between the nitrogen of proline and acyl group of other amino acids. Hydrolysis of this bond in dipeptides released from proteins, and thus the final stage of protein degradation, is catalysed by cytoplasmic prolidase (Wilk et al., 2017). The importance of prolidase for supplementing proline during its shortage has been demonstrated on the example of biosynthesis of collagen, a protein containing high proline residue content (Shim et al., 2012; Szoka et al., 2015; Szoka et al., 2019).

The p53-dependent activation of proline oxidase is a well-known phenomenon. However, the oxidative metabolism of proline and the associated generation of ROS in cancer cells is relatively rarely described as a mechanism of action of exogenous compounds. Investigation of the role of proline oxidase in apoptosis is particularly important in the case of cancers with p53 dysfunction. Their examples in the uterus are p53-mutant endometrial cancers (such Ishikawa cells) which are characterized by poor outcomes and cervical cancers associated with HPV infection which frequently have downregulated p53 level and transcriptional activity (such HeLa cells) (St John et al., 2000; Bouaoun et al., 2016). In the present study, we assessed the cytotoxic activity of flavonoids isolated from birch buds in Ishikawa and HeLa cell lines. We then investigated the mechanism of cytotoxic activity of santin, a most promising compound, including the possible association with proline catabolism.

## 2 Materials and methods

### 2.1 Flavonoids included in the study

Apigenin and luteolin were purchased from Sigma-Aldrich. All other flavonoids used in this study were isolated from the buds of downy birch.

Buds of downy birch were collected on August 2015 from trees growing in a non-protected area of the Biebrza National Park in north-eastern Poland (53° 32′ N, 22° 43′ E). Collection of buds was carried out in accordance with the local law. Permission was granted by the local forest inspectorate. The birch species was identified by Jolanta Nazaruk. Then, a method based on the nuclear DNA isolation and sequencing was used to confirm the identification of birch species (Isidorov et al., 2021). Voucher specimen (No. BO-17035) was deposited in the herbarium of the Department of Pharmacognosy, Medical University of Bialystok (Poland). All methods were carried out in accordance with relevant guidelines and regulations. Buds were kept at −18 C before use.

Carbon dioxide supercritical extraction of birch buds, as well as the subsequent isolation of individual flavonoids from the resulting extract by column chromatography and their identification using mass spectrometry and NMR, are described in our previous publications (Isidorov et al., 2018; Isidorov et al., 2021; Szoka et al., 2021).

### 2.2 Cell culture and cell treatment

Endometrial adenocarcinoma cells (Ishikawa) were purchased from Sigma-Aldrich. Cervix adenocarcinoma cells (HeLa), dermal fibroblasts (CCD25Sk) and breast epithelial cells (MCF 10A) were obtained from the American Type Culture Collection. Cancer cells and fibroblasts were cultured in Dulbecco’s Modified Eagle Medium supplemented with 10% foetal bovine serum and 1% penicillin/streptomycin (Gibco) in a 5% CO_2_ incubator at 37 C. MCF 10Acells were cultured in DMEM/F12 supplemented with 5% horse serum, 20 ng/mL epidermal growth factor, 500 μg/mL hydrocortisone, 100 ng/mL cholera toxin, 10 μg/mL insulin and 1% penicillin/streptomycin under the conditions given above. Santin was dissolved in DMSO, obtaining a concentration of 100 mM. The other flavonoids were also dissolved in DMSO, obtaining concentrations of 50 or 100 mM. The stock solutions were stored at −20 C. The working solutions (1 mM) were obtained by diluting the stock solutions with culture medium. A solution of DMSO in culture medium at a concentration corresponding to the highest santin concentration in each assay (0.025%–0.1%) was used as a control.

### 2.3 siRNA transfections

The siRNAs against p53 (assay ID s607), prolidase (assay ID s10299), proline oxidase (assay ID s11219), and negative control siRNA (Silencer Select Negative Control 1, 4390843) were purchased from Ambion. Ishikawa and HeLa cells were plated in 24-well plates (for cytotoxicity assay) or in 6-well plates (for obtaining homogenates) and allowed to adhere for 24 h. Then, the cells were transfected using Lipofectamine RNAiMax (Invitrogen, 13778100) according to the manufacturer’s protocol. After 24 h of treatment with siRNAs at final concentrations of 40 nM, the cells were immediately used for further experiments.

### 2.4 Cell viability assay—MTT assay

Cells were plated in 96-well plates at 1 × 10^4^ cells per well and allowed to adhere for 24 h. Cells were treated with flavonoids at concentrations of 3.1, 6.2, 12.5, 25, 50, and 100 μM and incubated for 24 or 48 h. MTT (3-(4,5-dimethyl-2-thiazolyl)-2,5-diphenyl-2H-tetrazolium bromide, M5655, Sigma-Aldrich) solution was added to each well and cells were incubated at 37 C for 4 h. The medium was then removed, and formazan crystals were dissolved in 100 μL of DMSO and 12.5 μL of Sorensen’s glycine buffer on a plate shaker. Absorbance was measured at 570 nm using a microplate reader. IC_50_ values were calculated using GraphPad Prism software.

### 2.5 Cell viability assay—neutral red uptake assay

Cells were plated in 96-well plates at 1 × 10^4^ cells per well and allowed to adhere for 24 h. Cells were treated with flavonoids at concentrations of 3.1, 6.2, 12.5, 25, 50, and 100 μM and incubated for 24 or 48 h. Then neutral red uptake assay was performed according to protocol (Repetto et al., 2008). Briefly, the culture medium was replaced by the solution of neutral red (72210, Sigma-Aldrich) in medium at a concentration 40 μg/mL. Plates were incubated at 37 C for 2 h. The neutral red solution was then discarded, and neutral red absorbed by cells was dissolved in 50% ethanol containing 1% glacial acetic acid. Absorbance was measured at 540 nm using a microplate reader and IC_50_ values were calculated using GraphPad Prism software.

### 2.6 Colony formation assay

In total, 250 cells were plated in each well of 12-well plates and allowed to adhere for 24 h. Cells were treated with santin at concentrations of 1.5, 3.1, 6.2, 12.5, and 25 μM for 48 h. Medium was discarded and fresh medium was added. After 7 days, culture medium was removed, and cells were washed once with phosphate-buffered saline (PBS, Gibco). Then, cells were fixed with 4% formaldehyde and stained with 0.1% crystal violet. Once colonies are stained, photographs of wells were taken using an imager and colonies were counted using ImageJ software (National Institutes of Health).

### 2.7 Apoptosis assay

Cells were seeded at a density of 1 × 10^5^ cells per well in 6-well plates and allowed to adhere for 24 h. Cells were treated with the respective concentration of flavonoids for 48 h. Floating and adherent cells were collected and assayed using a Dead Cell Apoptosis Kit with annexin V-fluorescein isothiocyanate (FITC) and propidium iodide (PI) for flow cytometry (#V13242, Thermo Fisher Scientific), according to the manufacturer’s protocol. Briefly, cells were dispersed in 100 μL annexin-binding buffer containing 5 μL annexin V-FITC conjugate solution, 1 μg/mL PI and 1 μg/mL Hoechst 33342 and incubated for 15 min at room temperature. Then 400 μL of annexin-binding buffer was added, and the cells were then analyzed with a DxFLEX flow cytometer (Beckman Coulter).

### 2.8 Western immunoblot

Adherent and floating cells were harvested and sonicated. Obtained homogenates were supplemented with 1% protease and phosphatase inhibitor cocktail (#78440, Thermo Fisher Scientific). The Lowry assay was performed to quantify protein content in the homogenates. Proteins (20–40 μg) were resolved on 7.5%, 10%, or 12% SDS-PAGE gels using the Mini-Protean Tetra system (Bio-Rad). Proteins were transferred to nitrocellulose membranes (Bio-Rad) using the Mini Trans-Blot Cell wet blotting system (Bio-Rad). Membranes were blocked with 5% skim milk for 1 h at room temperature and probed overnight at 4°C with primary antibodies. The following antibodies purchased from Cell Signaling Technology were used: anti-caspase-9 antibody (#9508, 1:1,000), anti-caspase-8 antibody (#9746, 1:1,000), anti-caspase-3 antibody (#9662, 1:1,000), anti-caspase-7 antibody (#12827, 1:1,000), anti-PARP antibody (#9542, 1:1,000) and anti-cleaved PARP antibody (#5625, 1:1,000); anti-proline oxidase antibody (sc-376401, 1:1,000) and anti-p53 antibody (sc-126, 1:500) were obtained from Santa Cruz Biotechnology; anti-prolidase antibody (STJ27369, 1:1,000) was purchased from St John’s Laboratory and anti-actin antibody (#A2066, 1:2,000) was obtained from Sigma-Aldrich. After extensive washes, a secondary antibody solution in 5% skim milk (anti-mouse IgG-HRP, Sigma-Aldrich, #A9044, 1:5,000 or anti-rabbit IgG-HRP, Sigma-Aldrich, #A9169, 1:5000) was added for 1 h at room temperature. Membranes were incubated with ECL-HRP substrate (GE Healthcare), and the signal was detected using the BioSpectrum Imaging System (Ultra-Violet Products, Ltd.). Intensity of the bands was quantified by densitometric analysis using VisionWorks LS software (Ultra-Violet Products, Ltd.).

### 2.9 Determination of prolidase activity

The activity of prolidase in cell homogenates was determined according to the method of (Myara et al., 1982). Briefly, prolidase was activated by overnight incubation of homogenates with 1 mM MnCl_2_ at 37°C. Then, the substrate (Gly-Pro, 100 mM) was added to each sample, followed by incubation at 37°C for 1 h. The reaction was stopped with 0.45 mM trichloracetic acid and samples were then centrifuged at 15,000 × *g* for 10 min. After adding acetic acid and Chinard’s reagent to collected supernatant, the mixtures were incubated at 90°C for 10 min. Samples were allowed to come to room temperature and then absorbance was measured at 515 nm. Enzyme activity was reported as nanomoles of proline released from the synthetic substrate during 1 min/mg of total proteins of cell homogenate.

### 2.10 Analysis of intracellular ROS

The oxidation-sensitive dye, 2′, 7′-dichlorofluorescein diacetate (DCFDA, D6883, Sigma-Aldrich), was used to determine the formation of intracellular ROS. Assay is based on the diffusion of DCFDA into the cell, where it is deacetylated by cellular esterases to a non-fluorescent compound, which is later oxidised by ROS into highly fluorescent 2′, 7′-dichlorofluorescein. Cells were plated in 96-well clear bottom black plates at 1 × 10^4^ cells per well and allowed to adhere for 24 h. Cells were treated with santin at concentrations of 6.2, 12.5, and 25 μM for 1, 24, or 48 h. Culture medium was discarded and the cells were washed with PBS and incubated with 10 μM DCFDA at 37°C for 30 min. Cell images were taken with fluorescence microscope.

### 2.11 Immunofluorescence microscopy

Cells were plated in 96-well clear bottom black plates at 1 × 10^4^ cells per well and allowed to adhere for 24 h. Cells were treated with the respective concentration of flavonoids and incubated for 48 h. Medium was removed and cells were fixed at room temperature for 15 min with 4% paraformaldehyde, rinsed three times with PBS, and permeabilised at room temperature for 5 min with 0.1% Triton X-100. Then, non-specific binding was blocked with 10% heat-inactivated goat serum in PBS for 1 h at room temperature. Cells were incubated with the following antibodies at 4°C, overnight: anti-p53 antibody (sc-126, 1:200) and anti-Nrf2 antibody (sc-365949, 1:200) obtained from Santa Cruz Biotechnology, and anti-cleaved-caspase-3 antibody (#9664, 1:200) and anti-cleaved-caspase-7 antibody (#8438, 1:200) purchased from Cell Signaling Technology. After three washes with PBS, cells were incubated with Alexa Fluor 594 conjugated antibody (A11032 or A11037; Invitrogen) for 1 h in the dark. Cell nuclei were stained with Hoechst 33342 (Invitrogen) and F-actin was stained with Phalloidin-Atto 488 (Sigma-Aldrich). Fluorescent signals were examined and captured using a BD Pathway 855 confocal microscope (Becton Dickinson). The mean fluorescence intensity was calculated using ImageJ software (National Institutes of Health).

### 2.12 Statistical analysis

Data were analysed in GraphPad Prism software using a one-way ANOVA followed by Tukey’s test, and reported as mean ± standard deviation (SD). For comparisons between two groups, two-tailed unpaired Student’s t-test was used. Values of *p* < 0.05 were considered to be statistically significant.

## 3 Results

### 3.1 Comparative cytotoxicity of birch bud flavonoids

**FIGURE 2:**
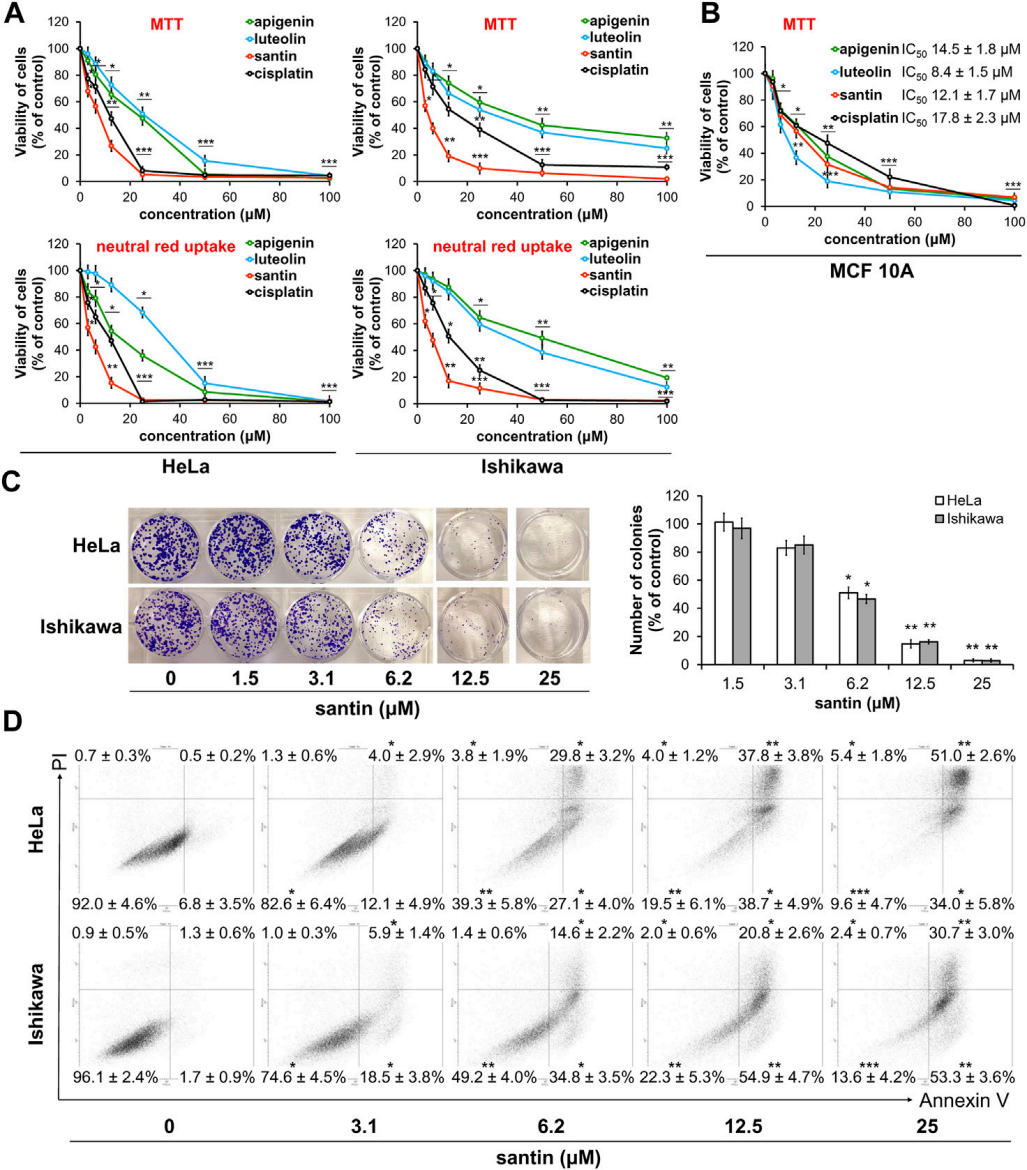
Santin decreases cell viability by induction of apoptosis. **(A)** Viability of HeLa and Ishikawa cells treated with santin at 48 h was evaluated by MTT assay and neutral red uptake assay. **(B)** Viability of MCF 10A cells treated with santin during 48 h was evaluated by MTT assay. **(C)** Colony formation assay of HeLa and Ishikawa cells treated with santin for 48 h and then cultured in fresh medium for 7 days. **(D)** HeLa and Ishikawa cancer cells were treated with santin for 48 h. Cells were co-stained with annexin V and propidium iodide. The percentage of apoptotic cells was determined using flow cytometry. Data are presented as mean ± standard deviation from three independent experiments done in triplicate. *p < 0.05, **p < 0.01 and ***p < 0.001 compared to the 0.1% DMSO-treated group (in MTT and neutral red uptake assays) or 0.025% DMSO-treated group (in colony formation and apoptosis assays).

Cytotoxic activity of the ten flavonoids isolated from birch buds was determined in cervical adenocarcinoma HeLa cells, endometrial adenocarcinoma Ishikawa cells, and in normal fibroblasts. Scheme of flavonoids isolation and chemical structures of compounds were shown in Supplementary Figure S1. Two of the flavonoids tested, cirsimaritin and apigenin 7,4′-O-dimethyl ether, belongs to flavones; another two, sakuranetin and naringenin 7,4′-O-dimethyl ether, are flavanones; and, the remaining six, ermanin, kaempferole, kumatakenin, quercetin, rhamnocitrin, and santin (5,7-dihydroxy-3,6,4′-trimethoxyflavone), belongs to flavanols. Two flavones, apigenin and luteolin, and the well-known chemotherapy drug, cisplatin, were used as reference agents. Viability of cells were assessed by two methods, MTT assay and neutral red uptake assay. The second method was used to eliminate potential incompatibility of flavonoids with MTT assay due to their reducing activity. Cells were incubated with flavonoids for 24 and 48 h and IC_50_ values were calculated. The IC_50_ values lower than 50 μM are presented in Table 1. We found that only two compounds, apigenin 7,4′-O-dimethyl ether and kumatakenin, significantly decreased viability of fibroblasts. Among the other eight flavonoids, santin was the most cytotoxic in both cancer cell lines. The effect of treatment with santin and reference agents for 48 h on cancer cells viability was shown in Figure 2A. The IC_50_ values for santin evaluated by MTT assay were 17.8 ± 2.0 μM (24 h) and 7.0 ± 0.8 μM (48 h) in HeLa cells, and 12.9 ± 1.1 μM (24 h) and 5.1 ± 0.9 μM (48 h) in Ishikawa cells. The IC_50_ values evaluated by neutral red uptake assay were generally lower than assessed by MTT assay and amounted 5.5 ± 1.3 μM (24 h) and 5.4 ± 0.8 μM (48 h) in HeLa cells, and 6.2 ± 1.0 μM (24 h) and 5.9 ± 1.1 μM (48 h) in Ishikawa cells. Among reference agents, apigenin was more cytotoxic than luteolin in HeLa cells. IC_50_ values for apigenin in HeLa cells were 30.0 ± 1.7 μM (24 h) and 17.9 ± 1.5 μM (48 h) for MTT assay, and 27.0 ± 1.9 μM (24 h) and 14.2 ± 1.1 μM (48 h) for neutral red uptake assay. Luteolin was more cytotoxic than apigenin in Ishikawa cells; however, only after 48 h of treatment IC_50_ values were lower than 50 μM and amounted to 25.9 ± 2.5 μM (MTT assay) and 31.6 ± 2.9 μM (neutral red uptake assay). Cisplatin reduced cell viability with IC_50_ values 30.0 ± 2.7 μM (24 h) and 9.4 ± 1.3 μM (48 h) in HeLa cells, and >50 μM (24 h) and 12.0 ± 2.0 μM (48 h) in Ishikawa cells, evaluated in MTT assay. The IC_50_ values of cisplatin evaluated by neutral red uptake assay were 20.0 ± 2.7 μM (24 h) and 9.7 ± 2.3 μM (48 h) in HeLa cells, and 28.1 ± 2.0 μM (24 h) and 11.7 ± 2.2 μM (48 h) in Ishikawa cells. Among seven other flavonoids from birch buds, cytotoxic activity of cirsimaritin was similar or even slightly higher than the activity of apigenin and luteolin. The IC_50_ values for cirsimaritin, evaluated by MTT assay, were 36.6 ± 2.2 μM (24 h) and 16.0 ± 1.9 μM (48 h) in HeLa cells, and >50 μM (24 h) and 19.6 ± 2.6 μM (48 h) in Ishikawa cells. The IC_50_ values for cirsimaritin, determined by neutral red uptake assay, were 17.5 ± 2.4 μM (24 h) and 13.9 ± 1.4 μM (48 h) in HeLa cells, and 34.2 ± 2.7 μM (24 h) and 26.1 ± 2.1 μM (48 h) in Ishikawa cells. In contrast, naringenin 7,4′-O-dimethyl ether and sakuranetin had little effect on viability of cancer cells (IC_50_ > 50 μM).

**TABLE 1:** IC_50_ values (±SD) of the birch flavonoids and reference agents in cervical adenocarcinoma HeLa cells, endometrial adenocarcinoma Ishikawa cells, and normal fibroblasts evaluated by MTT assay and neutral red uptake assay (IC_50_ values lower than 50 μM are shown).

IC_50_ values ± SD (μM) in MTT assay						
	HeLa		Ishikawa		Fibroblasts	
	24 h	48 h	24 h	48 h	24 h	48 h
apigenin 7,4′-O-dimethyl ether	—	—	26.2 ± 1.9	20.9 ± 1.4	18.0 ± 3.9	10.9 ± 4.6
cirsimaritin	36.6 ± 2.2	16.0 ± 1.9	—	19.6 ± 2.6	—	—
ermanin	—	31.8 ± 2.4	—	—	—	—
kaempferol	—	—	—	—	—	—
kumatakenin	—	—	30.5 ± 2.0	24.2 ± 1.8	47.0 ± 5.6	42.9 ± 4.3
naringenin 7,4′-O-dimethyl ether	—	—	—	—	—	—
quercetin	—	—	—	45.4 ± 4.1	—	—
rhamnocitrin	—	37.0 ± 2.1	—	42.5 ± 2.6	—	—
sakuranetin	—	—	—	—	—	—
santin	17.8 ± 2.0	7.0 ± 0.8	12.9 ± 1.1	5.1 ± 0.9	—	—
apigenin	30.0 ± 1.7	17.9 ± 1.5	—	33.1 ± 2.2	—	—
luteolin	42.8 ± 3.6	21.7 ± 1.3	—	25.9 ± 2.5	—	—
cisplatin	30.0 ± 2.7	9.4 ± 1.3	—	12.0 ± 2.0	—	40.5 ± 4.3

IC_50_ values ± SD (μM) in neutral red uptake assay						
	HeLa		Ishikawa		Fibroblasts	
	24 h	48 h	24 h	48 h	24 h	48 h
apigenin 7,4′-O-dimethyl ether	—	—	—	—	27.6 ± 2.8	7.6 ± 1.1
cirsimaritin	17.5 ± 2.4	13.9 ± 1.4	34.2 ± 2.7	26.1 ± 2.1	—	—
ermanin	39.0 ± 2.9	33.2 ± 1.9	—	46.7 ± 3.2	—	—
kaempferol	—	38.3 ± 2.5	—	—	—	—
kumatakenin	—	—	—	—	35.7 ± 2.5	15.3 ± 2.3
naringenin 7,4′-O-dimethyl ether	—	—	—	—	—	—
quercetin	39.1 ± 3.2	36.9 ± 2.0	—	42.9 ± 3.8	—	—
rhamnocitrin	—	41.2 ± 3.5	—	41.7 ± 2.5	—	—
sakuranetin	—	—	—	—	—	—
santin	5.5 ± 1.3	5.4 ± 0.8	6.2 ± 1.0	5.9 ± 1.1	—	—
apigenin	27.0 ± 1.9	14.2 ± 1.1	—	38.0 ± 2.1	—	48.6 ± 3.1
luteolin	31.5 ± 2.5	28.0 ± 1.5	—	31.6 ± 2.9	—	—
cisplatin	20.0 ± 2.7	9.7 ± 2.3	28.1 ± 2.0	11.7 ± 2.2	—	43.1 ± 3.5

To further evaluate cytotoxicity of santin on normal cells, breast epithelial cells MCF-10A were used. Cells were incubated with santin and reference agents for 48 h. As shown in Figure 2B all tested compounds decreased the viability of epithelial cells more efficiently than fibroblasts. In MTT assay IC_50_ value for santin was 12.1 ± 1.7 μM whereas IC_50_ values for apigenin, luteolin and cisplatin were 14.5 ± 1.8 μM, 8.4 ± 1.5 μM and 17.8 ± 2.3 μM, respectively.

These data indicate that santin has superior cytotoxicity than other birch bud flavonoids and both reference agents in cancer cells. The efficacy of flavonoids is time-dependent and increased incubation time results in lower cell survival rates. Generally, IC_50_ values obtained by MTT method was higher than those obtained by neutral red assay.

To confirm the high cytotoxicity of santin in HeLa and Ishikawa cells, we performed a colony formation assay (Figure 2C). HeLa and Ishikawa cells were treated with santin for 48 h and then cells were incubated with fresh medium for 1 week. Santin at 6.2 μM decreased the number of HeLa colonies by 49% and decreased number of Ishikawa colonies by 53%. Santin at 12.5 μM decreased the number of HeLa and Ishikawa colonies by 85% and 84%, respectively. These results are similar to those obtained in cell viability assays.

### 3.2 Santin activates extrinsic and intrinsic pathways of apoptosis

Dual staining with annexin V and propidium iodide was used to determine whether observed reduction of cell viability by santin is a result of apoptosis. As shown in Figure 2D, santin at a concentration of 6.2 μM significantly increased percentage of both early apoptotic cells to 27% ± 4% (HeLa) and 35% ± 4% (Ishikawa), and late apoptotic cells to 30% ± 3% (HeLa) and 15% ± 2% (Ishikawa). Treatment of cells with santin at a concentration of 12.5 μM resulted in further increase in the percentage of early apoptotic cells to 39% ± 5% (HeLa) and 55% ± 5% (Ishikawa). Percentage of late apoptotic cells was also increased to 38% ± 4% (HeLa) and 21% ± 3% (Ishikawa).

**FIGURE 3:**
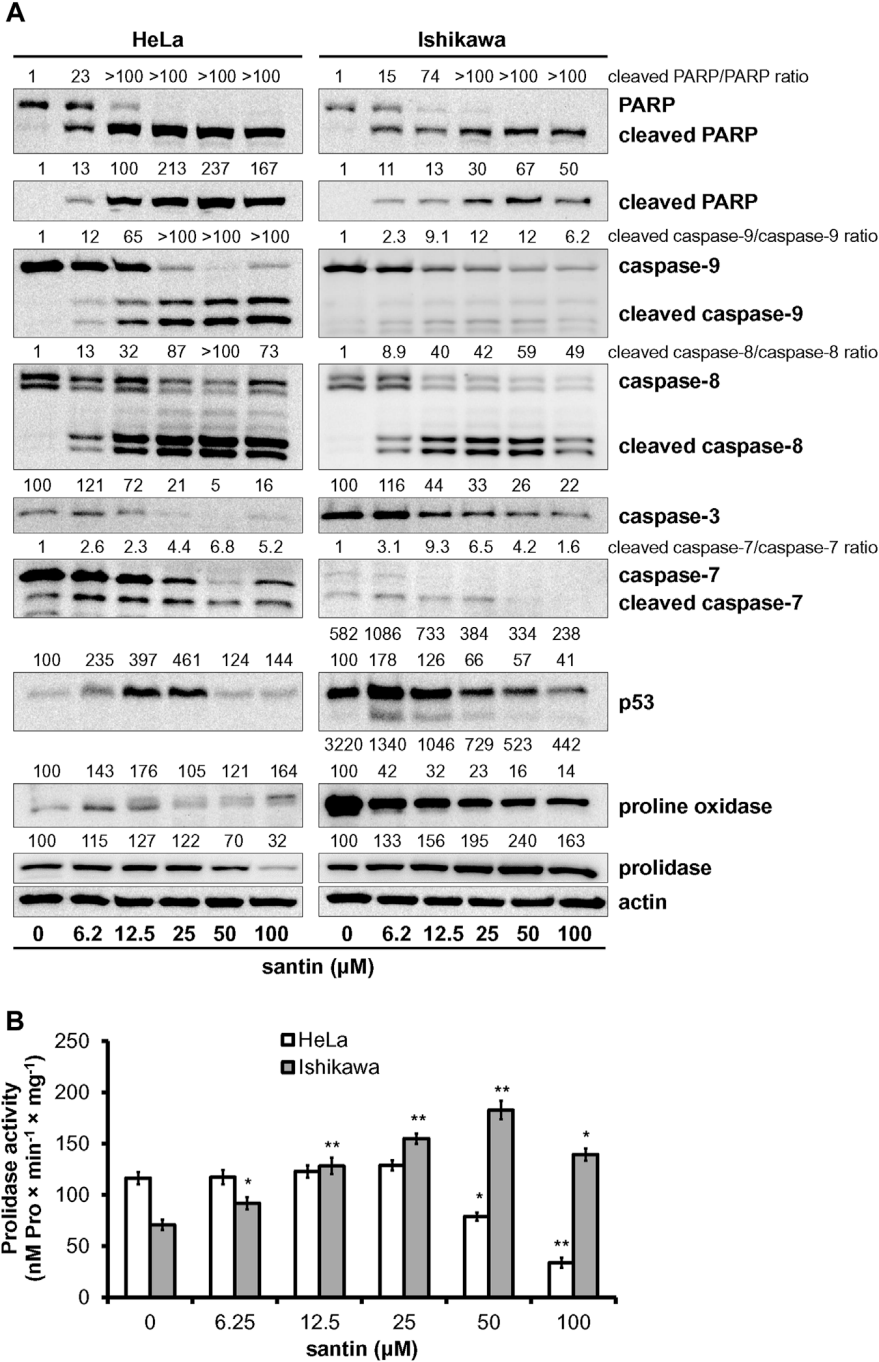
Santin induces intrinsic and extrinsic pathways of apoptosis and targets proline oxidase and prolidase. **(A)** Western blot analysis of PARP, caspase-9, caspase-8, caspase-3, caspase-7, p53, proline oxidase, and prolidase in HeLa and Ishikawa cells treated with santin for 48 h. Actin served as a control for protein loading. Control cells were treated with 0.1% DMSO. Results of densitometric analysis are located above the corresponding blots and presented vs. DMSO-treated cells of the corresponding cell line unless specified otherwise. If two rows of densitometric analysis results are given, the upper one presents results compared to DMSO-treated HeLa cells (HeLa 0 μM santin = 100 arbitrary units). The values are mean from three independent experiments normalized to the actin values. **(B)** Prolidase activity in HeLa and Ishikawa cells treated with santin for 48 h. The results present the mean values from three independent experiments ±SD. *p < 0.05 and **p < 0.01 compared to the control group (0.1% DMSO).

**FIGURE 4:**
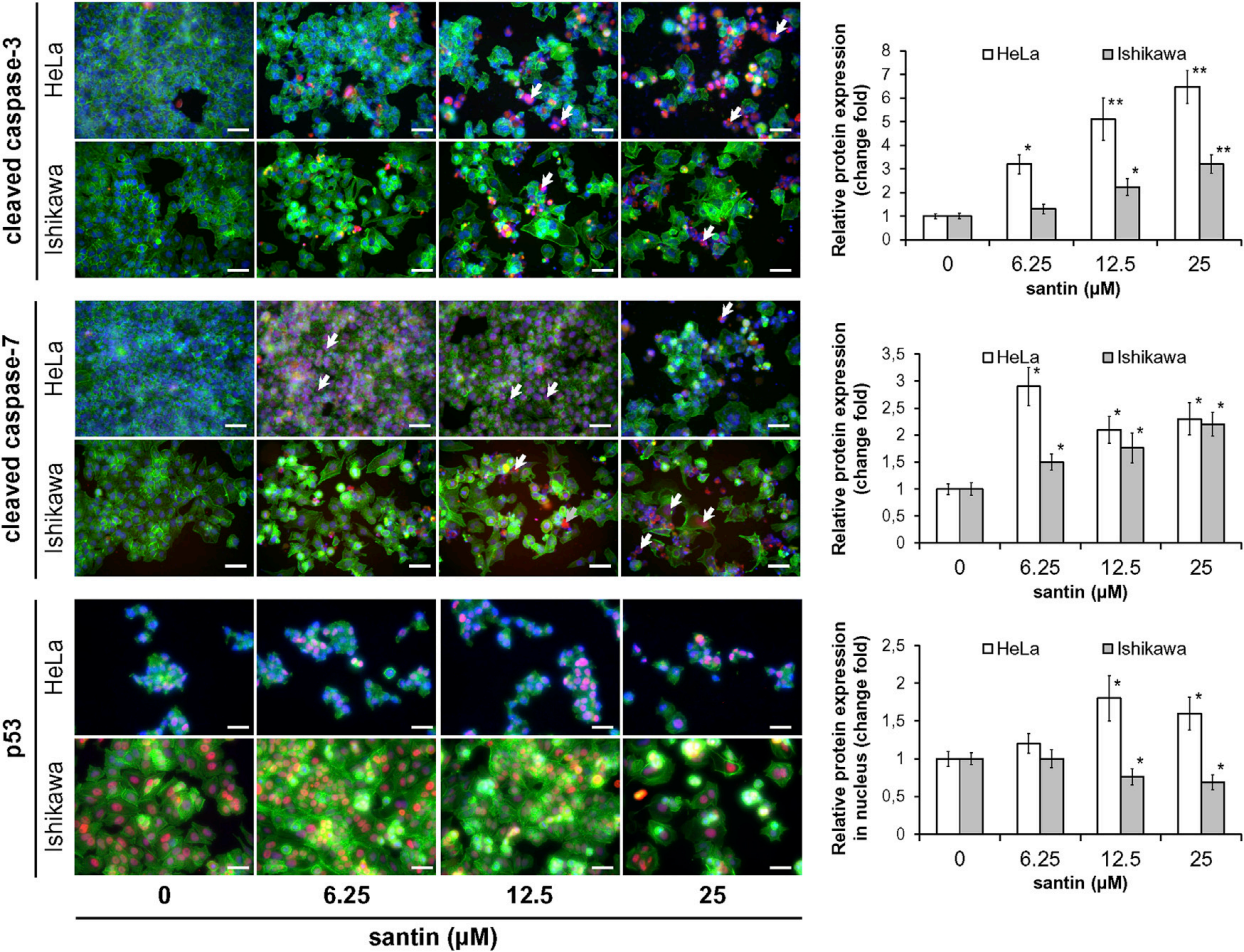
Santin activates effector caspases in HeLa and Ishikawa cells and induces nuclear accumulation of p53 in HeLa cells. Immunofluorescence imaging and quantification of cleaved caspase-3, cleaved caspase-7, and p53 expression (red fluorescence) in HeLa and Ishikawa cells treated with santin for 48 h. Cell nuclei were stained with Hoechst 33342 (blue fluorescence) and F-actin was stained with Phalloidin-Atto 488 (green fluorescence). Arrows point to few cells immunopositive for cleaved caspase-3 or cleaved caspase-7. Scale bar 50 μm. Objective ×20. *p < 0.05 and **p < 0.01 compared to the control group (0.025% DMSO).

To get insight into the mechanism of apoptosis induction by santin, level of key mediators of apoptosis was evaluated by Western blot. As shown in Figure 3A, treatment of HeLa and Ishikawa cells with santin for 48 h resulted in cleavage of both initiator caspases, caspase-9 and caspase-8, which was confirmed by a dose-dependent increase in cleaved forms and gradual decrease in the level of zymogens. Increase in expression of active initiator caspases was accompanied by a reduction in the level of zymogens of executor caspase-3 and caspase-7, and with an increase in the level of cleaved caspase-7. However, we did not obtain the signal specific for cleaved caspase-3 with immunoblot. Therefore, the expression of cleaved caspase-3 was detected by immunofluorescent staining (Figure 4). In both cell lines, treatment with santin led to an increase in the level of cleaved caspase-3. Immunofluorescence imaging also confirmed upregulated expression of cleaved caspase-7. Processing of executor caspases is accompanied by cleavage of the substrate of caspase-3, a poly(ADP-ribose) polymerase (PARP) as shown by Western blot (Figure 3A). We found that the p53 level in HeLa cells was significantly upregulated by santin in the range 6.2–25 μM, while in Ishikawa cells expression of p53 slightly increased in the range 6.2–12.5 μM and gradually decreased in a range 25–100 μM. Interestingly, both cell lines significantly differ in p53 protein basal level (expression of p53 is 5.8 times higher in Ishikawa cells as determined by densitometry). Increased p53 level in santin-treated HeLa cells was also showed by immunofluorescent staining (Figure 4).

Upregulation of p53 in HeLa cells treated with santin is accompanied by increased proline oxidase expression at concentrations of 6.2 and 12.5 μM, while in Ishikawa cells proline oxidase is downregulated (Figure 3A). Notably, the maximum level of proline oxidase in santin-treated HeLa cells (corresponding to 12.5 μM santin) is still 2.5 times lower than the minimum level of proline oxidase in Ishikawa cells (corresponding to 100 μM santin), as determined by densitometry. Santin downregulated prolidase expression in HeLa cells and increased its level in Ishikawa cells. The assessment of prolidase activity (Figure 3B) indicated that changes in expression of prolidase by santin affected enzyme activity. In HeLa cells, prolidase activity was downregulated and its minimum value was reached at 100 μM santin (decrease by 71%) while in Ishikawa cells, the activity increased significantly even at the lowest concentration of santin and reached its maximum at 50 μM (increase by 160%).

These results indicate that santin promotes apoptosis of HeLa and Ishikawa cells by intrinsic and extrinsic pathways. Notably, both an increase in proline oxidase expression in HeLa cells and an increase in prolidase activity while maintaining high proline oxidase expression in Ishikawa cells may lead to increased proline oxidation.

### 3.3 Generation of ROS is partly responsible for induction of apoptosis by santin

**FIGURE 5:**
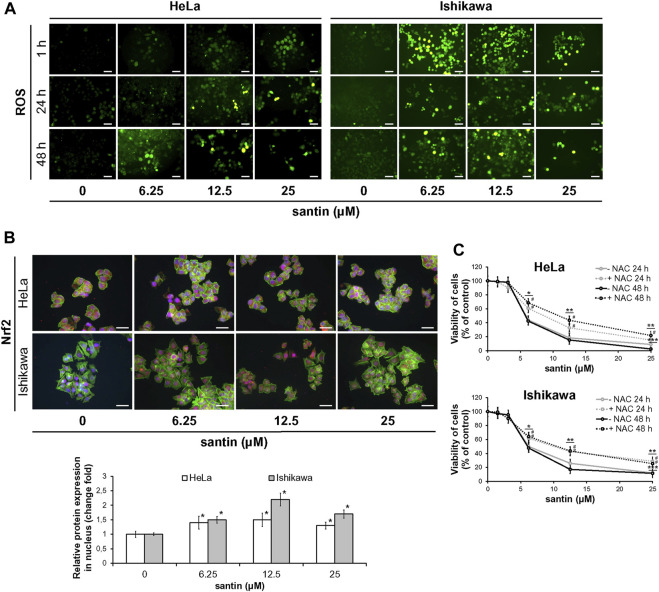
The reduction of cell viability by santin depends on ROS generation. **(A)** Fluorescence microscopy images of HeLa and Ishikawa cells treated with santin for 1, 24, and 48 h followed by staining with DCFDA. Control cells were treated with 0.025% DMSO. Scale bar 50 μm. Objective ×20. **(B)** Immunofluorescence imaging and quantification of Nrf2 expression (red fluorescence) in HeLa and Ishikawa cells treated with santin for 1 h. Cell nuclei were stained with Hoechst 33342 (blue fluorescence) and F-actin was stained with Phalloidin-Atto 488 (green fluorescence). Scale bar 50 μm. Objective ×20. *p < 0.05 compared to the control group (0.025% DMSO). **(C)** Viability of HeLa and Ishikawa cells preincubated with 2 mM N-acetyl-L-cysteine (NAC) for 2 h and then treated with various concentrations of santin for 48 h, evaluated by neutral red uptake assay. Data are presented as mean values from three independent experiments ±SD. *p < 0.05, **p < 0.01 and ***p < 0.001 compared to the control group (0.025% DMSO), # indicates *p* < 0.05 for pairwise comparison between plus or minus NAC.

Since oxidation of proline by proline oxidase is associated with generation of ROS, we next assessed whether santin affected the ROS level in HeLa and Ishikawa cells. Considering that ROS generation can occur quickly and may be transient, three time points were used (1, 24, and 48 h). The cells were treated with santin followed by incubation with general ROS probe DCFDA. As shown in Figure 5A, even short treatment with santin (1 h) resulted in massive ROS generation in Ishikawa cells and a significant increase in HeLa cells. With longer incubation times, santin upregulated ROS level in both cell lines; however, it was still more pronounced in Ishikawa cells. To confirm these results, we performed Nrf2 (nuclear factor erythroid 2-related factor 2) staining after treatment with santin for 1 h. As shown in Figure 5B, santin induced nuclear accumulation of Nrf2 in both cell lines; however, as in ROS analysis, Ishikawa cells were more affected. Immunofluorescence imaging also revealed that basal level of Nrf2 in HeLa cells is higher than in Ishikawa cells. To determine whether upregulated generation of ROS by santin is responsible for reduction of cell viability, we performed neutral red uptake assay in cells pre-treated with ROS scavenger N-acetyl-L-cysteine (NAC). As shown in Figure 5C, NAC pre-treatment attenuated cytotoxicity of santin in both cell lines, however, the effect was more pronounced in Ishikawa cells. The IC_50_ values in HeLa cells were 18.2 ± 2.0 μM (24 h, without NAC), 25.6 ± 2.4 μM (24 h, NAC treated), 6.9 ± 1.1 μM (48 h, without NAC), and 11.6 ± 1.4 μM (48 h, NAC treated); and in Ishikawa cells, IC_50_ values were 12.7 ± 1.3 μM (24 h, without NAC), 25.6 ± 2.1 μM (24 h, NAC treated), 4.8 ± 1.0 μM (48 h, without NAC), and 11.0 ± 1.4 μM (48 h, NAC treated).

Our data suggest that ROS generation is partly responsible for cytotoxicity of santin in HeLa and Ishikawa cells.

### 3.4 Prolidase and proline oxidase contributes to induction of apoptosis by santin in Ishikawa cells

**FIGURE 6:**
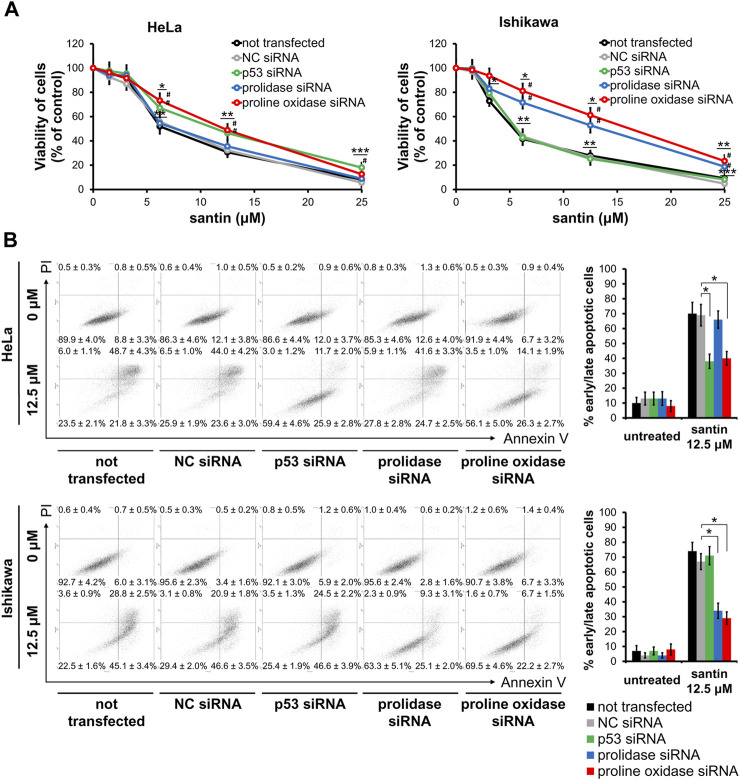
Decrease in cell viability induced by santin is partially prevented by silencing of p53 and proline oxidase in HeLa cells and silencing of prolidase and proline oxidase in Ishikawa cells. **(A)** Viability of HeLa and Ishikawa cells treated with siRNA followed by incubation with santin for 48 h evaluated by MTT assay. Data are presented as mean values from three independent experiments ± SD. *p < 0.05, **p < 0.01 and ***p < 0.001 when compared to the control group (0.025% DMSO), # indicates *p* < 0.05 compared to the NC siRNA-transfected cells. **(B)** HeLa and Ishikawa cells were treated with siRNA followed by incubation with 12.5 μM santin for 48 h. Cells were co-stained with annexin V and propidium iodide. Percentage of apoptotic cells was determined using flow cytometry. Data are showed as mean ± SD from three independent experiments. Control group was treated with 0.012% DMSO. *p < 0.05 compared to the NC siRNA-transfected cells.

**FIGURE 7:**
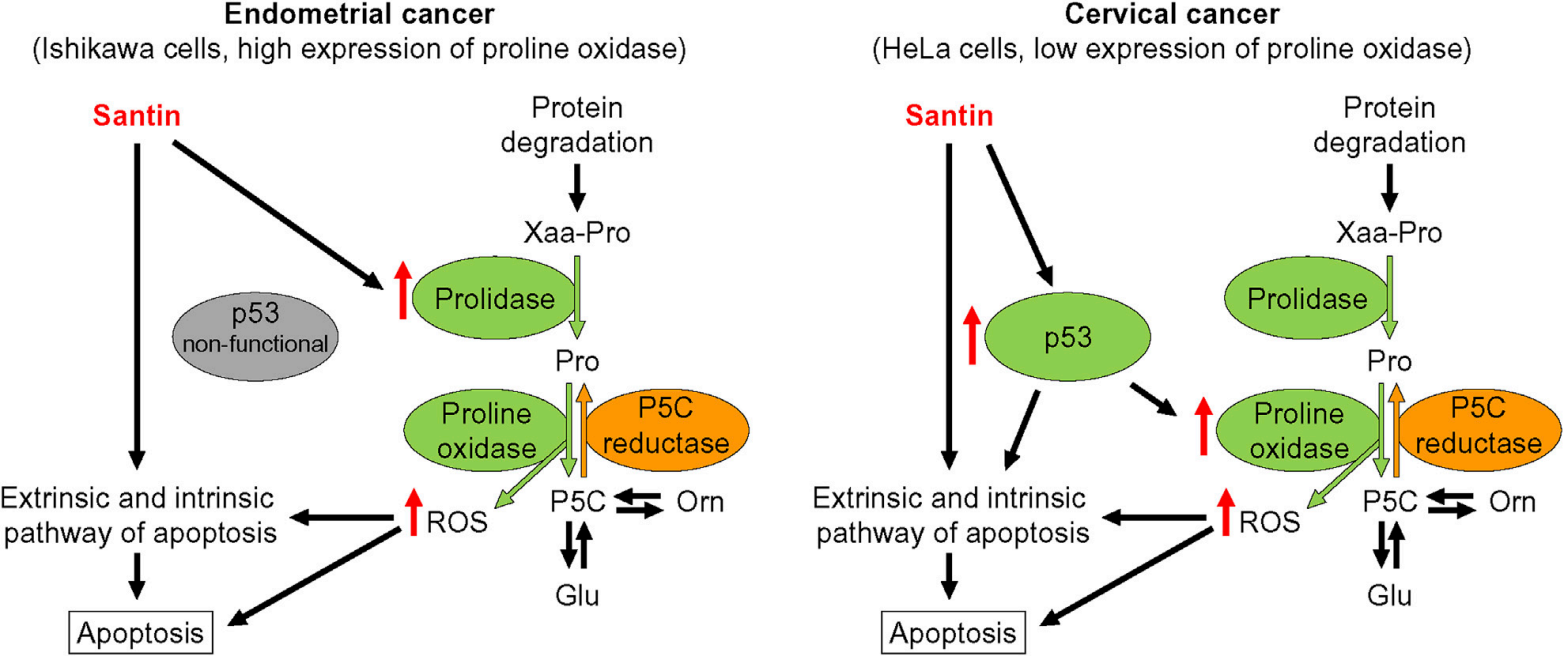
Proposed mechanisms of apoptosis induction by santin in endometrial cancer Ishikawa cells and cervical cancer HeLa cells. P5C, pyrroline-5-carboxylate.

We further investigated the role of proline oxidase, prolidase, and p53 in reduction of cell viability by santin. For this purpose, HeLa and Ishikawa cells were treated with siRNAs targeting proline oxidase, prolidase, and p53, or with negative control siRNA. The efficacy of protein silencing was determined by Western blot analysis (Supplementary Figure S2). Use of siRNAs resulted in significantly decreased expression of each targeted protein in both cell lines. Further, the cells were treated with santin for 48 h and cell viability was evaluated by MTT assay (Figure 6A). The IC_50_ values in santin-treated HeLa cells were 7.2 ± 0.9 μM in non-transfected cells, 7.3 ± 1.1 μM in negative control siRNA transfected cells, 10.9 ± 1.9 μM in p53-silenced cells, 7.8 ± 1.0 μM in prolidase-silenced cells, and 11.2 ± 1.3 μM in proline oxidase-silenced cells. The IC_50_ values in santin-treated Ishikawa cells were 5.7 ± 0.8 μM in non-transfected cells, 5.9 ± 0.9 μM in negative control siRNA transfected cells, 5.9 ± 1.1 μM in p53-silenced cells, 11.1 ± 1.9 μM in prolidase-silenced cells, and 14.0 ± 2.0 μM in proline oxidase-silenced cells. Assessment of apoptosis (Figure 6B) confirmed the results obtained by MTT assay. Apoptosis induced by 12.5 μM santin was significantly impaired in HeLa cells with knockdown p53 and proline oxidase while the similar effect was observed in Ishikawa cells with silenced prolidase and proline oxidase. The data suggest that p53 and proline oxidase contributes to santin-induced apoptosis of HeLa cells. In contrast, p53 is not engaged in Ishikawa cell apoptosis promoted by santin. Prolidase and proline oxidase, however, significantly contributes to santin-induced apoptosis of Ishikawa cells. Importantly, silencing of these proteins did not completely prevent santin cytotoxicity. Therefore, induction of apoptosis by santin is multifactorial and depends partly on proline catabolism (Figure 7).

## 4 Discussion

The ongoing fight against cancer requires the development of new drugs with strong targeted effects while keeping side effects as low as possible. The anticancer activity of flavonoids and their semisynthetic derivatives has been indicated for years. However, mechanisms by which flavonoids reduce cancer cell viability and promote apoptosis are not fully understood. Here we introduce santin, a flavonol exerting high cytotoxic activity in cervical cancer HeLa cells and endometrial cancer Ishikawa cells, and only slightly affecting viability of normal cells. Induction of apoptosis by santin depends partially on upregulated oxidative proline catabolism and generation of ROS.

Viability of cells is commonly determined by colorimetric assays. MTT assay is based on reduction of MTT catalysed by flavin-containing mitochondrial reductases as well as NAD(P)H-dependent oxidoreductases and dehydrogenases (Berridge et al., 2005; Jo et al., 2015). Reducing compounds such as certain flavonoids were found to interfere with the MTT test (Peng et al., 2005). In contrast, neutral red uptake assay principle is that neutral red penetrates the cell membranes and is retained in lysosomes in protonated form. Thus, viable cells accumulate neutral red because they are able (through ATP production) to maintain pH of lysosomes lower than that of the cytoplasm (Repetto et al., 2008). And indeed, for some flavonoids, significant differences were obtained in the results of these two tests. Therefore, our results indicate the need to use at least two cytotoxicity assays to evaluate viability of cells treated with flavonoids.

Our data showed stronger reduction of HeLa and Ishikawa cells viability by santin than that induced by apigenin and luteolin. However, viability of HeLa cells treated with apigenin (IC_50_ values were around 30.0 μM after 24 h and 17.9 μM after 48 h, as determined by MTT test, and 27.0 μM after 24 h and 14.2 μM after 48 h, as determined by neutral red uptake assay) were reduced in lower extent at the 48 h time point than reported in another study (IC_50_ determined by MTT assay around 38.0 μM after 24 h and 10.0 μM after 48 h) (Souza et al., 2017). In contrast, cytotoxicity of luteolin at the 48 h time point (IC_50_ values were around 21.7 μM in MTT test and 28.0 μM in neutral red uptake assay) was similar to that obtained by others (20 μM luteolin induced about 50% reduction in HeLa cell viability, as determined by MTT assay) (Raina et al., 2021). The advantage in the cytotoxic activity of santin over apigenin was previously observed by us in cancer cells of the stomach, liver, and colon (Szoka et al., 2021). Moreover, reduction of normal cell viability by santin was similar to that induced by apigenin and luteolin. Thus, increased activity against cancer cells is not accompanied by an increase in toxic effects against normal cells, which is a desirable feature for potential anticancer drugs.

Numerous studies showed that promotion of apoptosis is responsible for impairing cancer cell viability by flavonoids (Csapi et al., 2010; Souza et al., 2017; Lei et al., 2019; Raina et al., 2021; Zhan and Wei, 2021; Liang et al., 2022). In our study, santin induced cleavage of caspase-8 and caspase-9, the common features of triggering extrinsic and intrinsic pathways, respectively. Induction of both apoptotic pathways by santin was previously reported in digestive system cancer cells (Szoka et al., 2021). Moreover, activation of extrinsic pathway by increase in expression of TRAIL receptors (TRAIL-R1 and TRAIL-R2) was recently shown in santin treated colon cancer cells (Kłósek et al., 2023). Although, santin similarly affected apoptosis pathways in both cell lines, a divergent effect was found at the level of p53. Expression of p53 was upregulated in HeLa cells while it was downregulated in Ishikawa cells, which may suggest a differential effect of p53 on apoptosis in these cells. It can be explained on a functional level. Cervical cancer cell line HeLa is HPV-positive and shows low basal expression of p53 but the *TP53* gene is not mutated (St John et al., 2000). In contrast, endometrial cancer cell line Ishikawa carries missense mutations (D49H and M246 V) in the *TP53* gene. Although D49H was shown to not impair activity of p53 (Devor et al., 2020), M246 V mutation is located in the part of the crucial Zn^2+^ binding site which may have a consequence on the DNA-binding capacity (Doffe et al., 2021). And in fact, it was reported about loss of p53 function in Ishikawa cells (Bouaoun et al., 2016). In contrast to the HeLa cells, p53 basal level in the Ishikawa cells is high and it is probably due to presence of M246 V mutation (Doffe et al., 2021).

It is worth noting that p53 reactivation by attenuating E6-dependent p53 degradation is indicated as a mechanism for the induction of apoptosis by certain flavonoids in HPV-positive cervical cancer cells (Cherry et al., 2013). For instance, flavone chrysin was found to increase the level of ROS and thus to upregulate p53 expression and induce its translocation to the nucleus for triggering apoptosis (Pawar et al., 2022). In our study, treatment of HeLa cells with santin resulted in upregulation of p53, as well as its nuclear accumulation, and it was accompanied by an increase in proline oxidase expression. Importantly, p53 is a well-known inducer of proline oxidase gene transcription (Polyak et al., 1997), which indicates that an increase in p53 expression by santin is accompanied by an increase in its transcriptional activity. It is supported by attenuation of santin-induced apoptosis of HeLa cells by p53 silencing. Moreover, silencing of proline oxidase indicated the pivotal role of proline oxidase as executor protein for p53-dependent apoptosis, since silencing both p53 and proline oxidase resulted in similar attenuation of santin-induced reduction of cell viability. Therefore, santin partly reactivates p53 to promote apoptosis in HeLa cells.

In contrast to the data obtained in HeLa cells, it was surprising that reduction of p53 expression by santin in the Ishikawa cell line was accompanied by decrease in proline oxidase level. However, level of proline oxidase was not affected by silencing of p53, which confirms that there is no relationship between the expression of these two proteins. Considering lack of p53 activity, high expression of proline oxidase in Ishikawa cells indicates another unknown factor responsible for such high expression. Its identification would be beneficial for further research on proline-dependent apoptosis in cancer cells. Our data raised a question whether proline oxidase, whose level is decreased by santin, might really contribute to apoptosis induction. It seems that a decrease in expression of proline oxidase is not important, but rather keeping its high level despite this reduction.

The presence of wild type p53 is not required for proline oxidase-dependent apoptosis (Maxwell and Davis, 2000) but the enzyme must have access to its substrate (proline) to generate ROS followed by caspase-9 activation (Hu et al., 2007). The main source of proline in normal cells is glutamine, which after removing amide nitrogen form glutamate, a direct precursor of proline. The role of glutamine in proline biosynthesis is particularly evident in cultured cells, growing in glutamine abundant medium, for instance in fibroblasts. Hallmark of fibroblasts is a very low level of proline oxidase (around 40 times lower than in Ishikawa cells) that directs proline to biosynthesis of extracellular matrix components (mainly collagen) but not to proline oxidase-catalyzed degradation (Szoka et al., 2017). However, rapid growth of cancer cells requires metabolic adaptation to provide large amounts of amino acids for protein synthesis (Zhang et al., 2017). In that case, biosynthesis of endogenous amino acids may not meet the demand and consequently impair cell proliferation (Maddocks et al., 2017; Knott et al., 2018). Therefore, recycling of amino acids from protein breakdown may become a solution for this deficiency (Davidson et al., 2017). This may become crucial for proline supply since cancer cells are characterised by proline shortage that restricts protein synthesis (Loayza-Puch et al., 2016). Our data showed that santin in concentration close to its IC_50_ significantly increased expression and activity of prolidase, an enzyme catalysing the final step in the recovery of proline from proteins. Induction of prolidase activity by certain flavonoids was also reported by us previously (Karna et al., 2011). The contribution of prolidase and proline oxidase in promotion of apoptosis by santin in Ishikawa cells was confirmed by using siRNA transfections. The important role of proline recycled by prolidase has been shown in both normal and cancer cells. In dermal fibroblasts, cells addicted to proline due to extensive biosynthesis of extracellular matrix components, induction of prolidase stimulated TGFβ signalling (Surazynski et al., 2010), and collagen biosynthesis (Shim et al., 2012; Szoka et al., 2019). In breast cancer, proline released by prolidase was found to regulate HIF-1α expression (Surazynski et al., 2013) and stimulate autophagy (Zareba et al., 2020). Thus, released proline under conditions of high amino acid demand can have a significant impact on cell metabolism and cell functions including apoptosis.

The increase in prolidase expression may contribute to apoptosis not only by enzymatic-dependent mechanism but may be also enzymatic-independent. Prolidase was reported to bind to the proline-rich domain in p53, suppressing its function (Yang et al., 2017). Moreover, missense mutations in the DNA-binding domain of p53 do not change its affinity to prolidase (Yang et al., 2021); however, the mutation present in Ishikawa cells has not been studied. Nevertheless, this observation may be important in the context of acquiring oncogenic function by mutant p53 (gain-on-function). It has been found that knockdown of mutant p53 inhibits the growth of cancer cells and promotes apoptosis (Zhu et al., 2013). However, in our study, silencing of p53 had no effect on Ishikawa cell viability compared with negative control siRNA-transfected cells. This is consistent with recent research, showing only mild reduction in viability of endometrial cancer cells after p53 silencing (Fares et al., 2021).

Flavonoids are well recognised antioxidants, however, in certain conditions they can induce oxidative stress and trigger apoptosis, especially in cancer cells (Souza et al., 2017). Treatment of Ishikawa and HeLa cells with santin led to apoptosis by increased ROS generation. Notably, ROS level in Ishikawa cells remained high even after prolonged incubation with santin despite rapid increase in Nrf2 protein expression and its accumulation in the nucleus. In normal conditions, Nrf2 is bound to Keap1 (Kelch ECH associating protein 1) that promotes proteasomal degradation of Nrf2. The bond between proteins is disrupted by ROS, and Nrf2 accumulates in the nucleus and subsequently activates gene transcription of multiple antioxidant enzymes (Ulasov et al., 2022). Our results indicate that antioxidant defense system activated in Ishikawa cells is not sufficient to remove ROS and thereby to avoid apoptosis induced by ROS.

Our findings indicate the involvement of proline oxidase in the induction of apoptosis in cervical and endometrial cancer cells. In the case of cervical cancer, this is due to reactivation of the p53 protein, while in the endometrial cancer is due to overexpression of prolidase providing proline for proline oxidase. To date, expression of proline oxidase was assessed in a relatively small number of cancers showing reduced expression in human cancer tissues, especially from the digestive tract and kidneys (Maxwell and Rivera, 2003; Liu et al., 2009). Therefore, characterising of the proline oxidase expression in endometrial cancer seems to be important since it is possible to maintain high expression of proline oxidase despite mutation in *TP53*. Finally, our study indicates induction of prolidase activity as an approach for proline oxidase-dependent apoptosis.

## 5 Conclusion

Our results showed that santin reduces viability of cervical cancer HeLa cells and endometrial cancer Ishikawa cells by induction of apoptosis. The decrease in cell viability induced by santin was partially prevented by the silencing of p53 and proline oxidase in HeLa cells and the silencing of prolidase and proline oxidase in Ishikawa cells. Therefore, induction of apoptosis by santin in endometrial cancer cell line depends partly on increased recycling of proline by prolidase and its subsequent oxidation by proline oxidase. However, further *in vivo* studies are needed to assess anti-cancer activity of santin and to fully understand its mechanism of action.

## Data Availability

The raw data supporting the conclusion of this article will be made available by the authors, without undue reservation.
