# Supplementary material for: Prolidase-proline oxidase axis is engaged in apoptosis induction by birch buds flavonol santin in endometrial adenocarcinoma cell line
Source: Front Mol Biosci. 2023 Sep 6;10:1247536. doi: 10.3389/fmolb.2023.1247536 (PMC10512030; doi:10.3389/fmolb.2023.1247536)
Supplement: Supplementary file 1 [file DataSheet1.pdf]

Supplementary Material

Prolidase-proline oxidase axis is engaged in apoptosis induction by birch buds flavonol santin in endometrial adenocarcinoma cell line

Lukasz Szoka*, Jolanta Nazaruk, Joanna Giegiel, Valery Isidorov

*** Correspondence:** Lukasz Szoka: lukasz.szoka@umb.edu.pl

1.1 Supplementary Figures

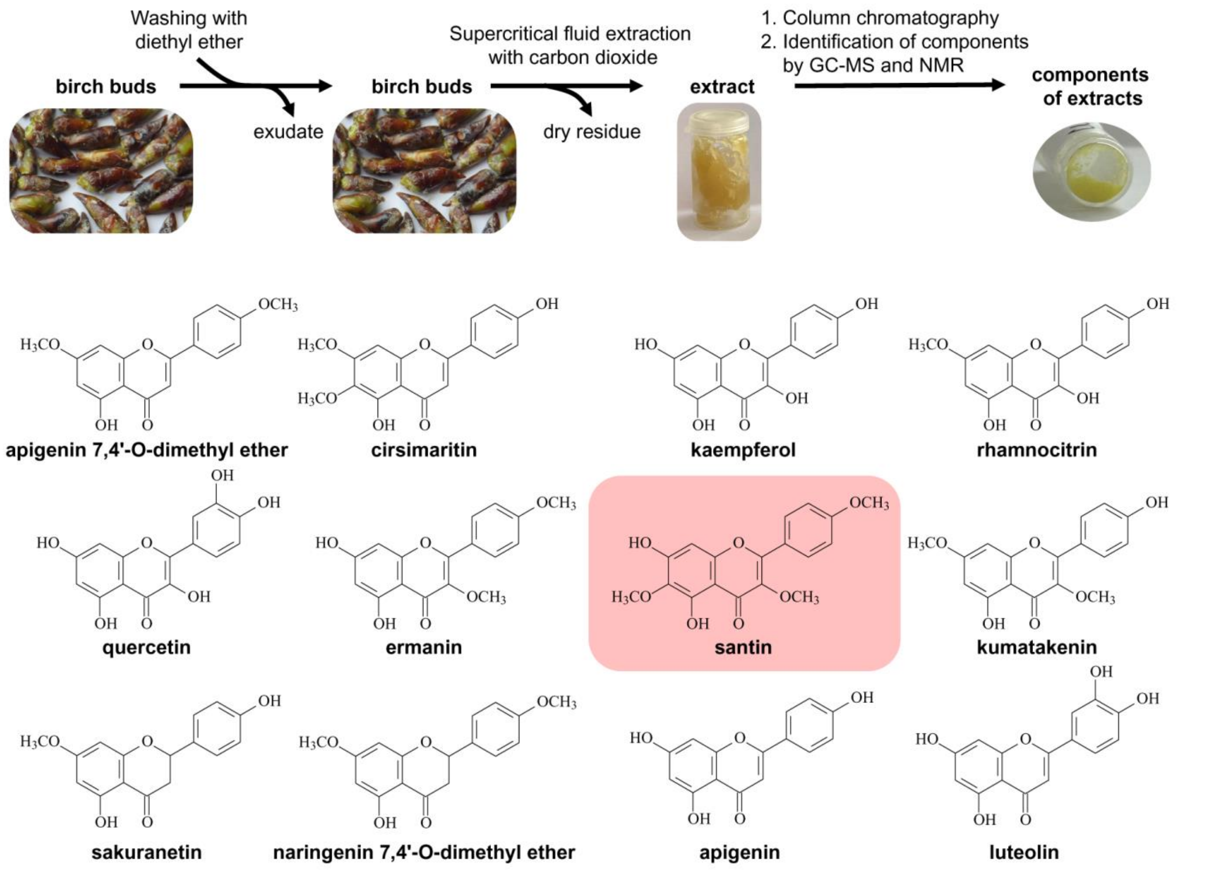

Supplementary Figure 1. Process of flavonoids isolation from downy birch buds and chemical structures of flavonoids used in the study.

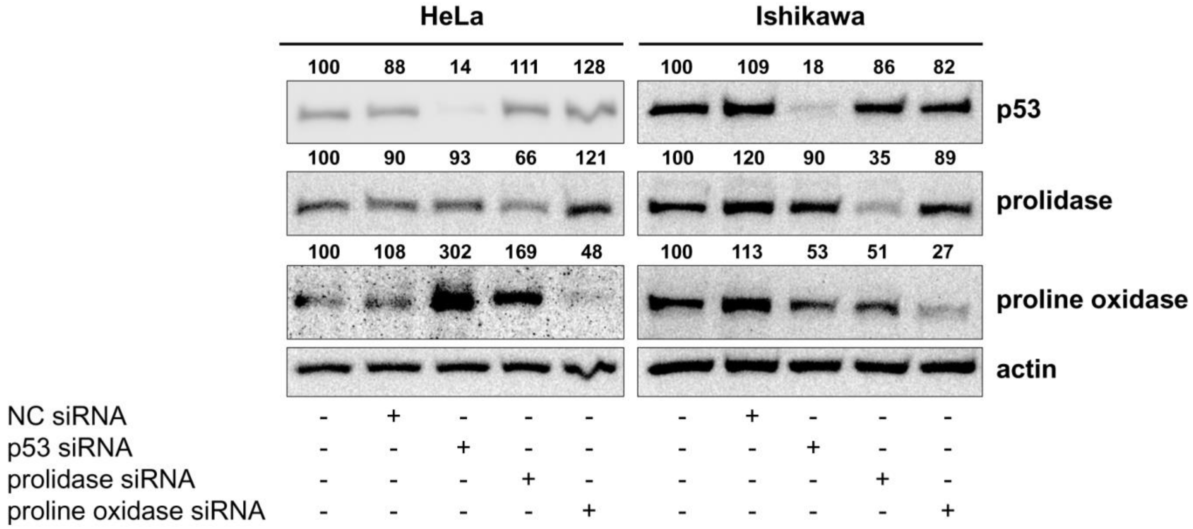

Supplementary Figure 2. Western blot analysis of p53, prolidase, and proline oxidase in HeLa and Ishikawa cells treated with siRNA targeting p53, prolidase, proline oxidase, or negative control (NC) siRNA. Actin served as a control for protein loading. Results of densitometric analysis are located above the corresponding blots and presented vs. non transfected cells. The values are mean from three independent experiments normalized to the actin values.
